# A balanced scorecard for assessing a strategic plan in a clinical laboratory

**DOI:** 10.11613/BM.2019.020601

**Published:** 2019-04-15

**Authors:** Luisa Alvarez, Anna Soler, Leonor Guiñón, Aurea Mira

**Affiliations:** 1 Quality Unit, Biomedical Diagnostic Center, Hospital Clínic, Barcelona, Spain; 2 Managing Director, Biomedical Diagnostic Center, Hospital Clínic, Barcelona, Spain

**Keywords:** balanced scorecard, clinical laboratory, management, strategic plan

## Abstract

The Balanced Scorecard (BSC) is a tool for strategic management that is used in many companies and organizations worldwide, both in the public and private sector. With this purpose it has also been used in healthcare organizations and institutions but there are not many studies on the implementation of BSC methodology in the day-to-day clinical laboratory. This review shows the strategy for the development of a BSC, which includes theoretical perspective objectives, as well as some indicators and goals with which the monitoring and quantitative measurement of the achievements of a strategic plan in a clinical laboratory can be done. Moreover, the results of the indicators allow the prioritization of the initiatives to be implemented each year. The methodology for the development of the proposed BSC includes the following steps: definition of theoretical objectives of each of the perspectives most used in the management of a clinical laboratory (customers, financial, internal processes and learning) taking into account the vision and the organizational model of the laboratory; creation of a strategic map of perspective objectives; definition of the relevant indicators to follow up on the objectives in a quantitative manner and establishment of the goals. Whether or not the laboratory is a reference laboratory, in which specific and infrequent analysis and health population programs are performed, is another fact to take into account. In this review a BSC for a reference clinical laboratory of the Spanish public sector is shown.

## Introduction

Many organizations still present the results obtained from the application of a strategic plan as a list of achievements reached, sometimes accompanied by budget compliance but without using any other type of analysis, which includes the study of the impact of their results on the whole. In this regard, a tool that can be used to track and obtain quantitative data of the degree of achievement of the strategic plan’s objectives is the Balanced Scorecard (BSC). The BSC is one of the most popular performance management tools, which categorizes the quantifiable objectives of an organization into four perspectives: financial, customer, learning and internal processes (*1*). For each objective, indicators and their goals are defined in order to provide objective and quantitative information about the achievements. It was introduced by Kaplan and Norton in the 1990 and, although it was originally used in industry, over time its use has been extended to applications beyond that of strategic management in this field (*2*, *3*). Shortly after its creation it was introduced for the measurement of performance in healthcare organizations and institutions in the USA, Canada and in Europe (*4*-*9*). Although the BSC was applied as a tool for strategic management in laboratory analysis as early as 2003, few studies have been developed since then (*10*). Among them there are those of Salinas, that use indicators of the laboratory processes classified according to the four perspectives cited and of Salas which orders the indicators according to the perspective of the internal process (*11*-*13*). It is worth highlighting this shortage of studies in Spain, despite the fact that in 2008, the Spanish Society of Laboratory Medicine (SEQCML) defined Recommendations for the development of a BSC in the clinical laboratory (*14*). Moreover, there are no studies on the implementation of its use to assess results of the application of a BSC for monitoring long-term strategic plans.

Given that the BSC’s usefulness for evaluating a strategic plan in hospital management has been shown, we hypothesized that the BSC would provide a useful tool for the Managing Board of clinical laboratories, both to describe the vision and strategy of their laboratories and to manage implementation and assess the achievements of a four-year strategic plan. Thus, the aim of this review is to show the strategy that should be followed for the development of a BSC, which includes perspective objectives as well as the indicators and goals with which the monitoring and quantitative control of the achievements can be carried out, in the short and long-term of a strategic plan.

## Strategy for the development of the BSC

The Managing Board has to take into account the vision of the clinical laboratory and the management model of the organizational structure when designing a BSC.

The strategy to use includes the following steps:

### 1. Definition of theoretical perspective objectives

These objectives arise from the answers to the questions that are formulated considering the four main perspectives that exist in the management of a laboratory’s activity:

**Customer perspective**: How to increase the value with which customers perceive our activity? What are their needs and expectations? As some authors have indicated, health institutions have to devote the greatest efforts to this perspective, since they are the ultimate recipients of the actions of improvement and the objectives defined in all perspectives (*8*, *15*). Recipients of the activity of the clinical laboratory are: citizens, patients and clinical physicians of the hospital. Furthermore, in the case of a reference clinical laboratory, physicians or users of other laboratories who send samples for analysis should be included. As well, in some clinical laboratories the Ministry of Health of a country’s Government, that commissions the laboratory population health programs, will be incorporated.

Among the objectives to take into account in this perspective is the improvement of patient safety in order to prevent patients from potential risks in all phases of the analytical process, as Plebani has shown (*16*). On the other hand, the clinical laboratories that have implemented the ISO 15189 standard, from the 2012 version have to demonstrate that they are managing the risks that can affect patient safety (*17*).

**Financial perspective**: How should providers of financial resources perceive us? How should the clinical laboratory achieve additional incomes to those of the Ministry of Health of a country’s Government? Taking into account the comments in the customer perspective, in healthcare organizations the objectives of this perspective should go after those of the customer’s perspective, as indicated by Kaplan and Norton.

**Internal process perspective**: In which processes should laboratory staff focus their efforts on and be excellent in satisfying their customers? Can an increase in efficacy and efficiency in these processes be shown?

**Learning perspective**: How to achieve a greater involvement of staff that encourages greater efficiency, higher quality and more innovation? It is an established fact that in all organizations it is necessary to manage the human factor to a high standard. Both the one that occupies strategic positions and the one directly in charge of performing the analysis, since this factor is what the organization is based on and that which allows the organization to achieve excellence. Professionals appreciate the fact that the Managing Board of a clinical laboratory is concerned with satisfying all those requirements that impact the work environment. Therefore, a large number of the objectives of the learning perspective must be aimed at enhancing the acquisition of greater skills for the personnel and their professional development, and improving their motivation and increasing satisfaction.

Finally, the objectives of the strategic plan are included in the theoretical perspective’s objectives. At this point, it has to be mentioned that real objectives of the strategic plan can have very different scopes, particularly in clinical laboratories that include the activity of all specialties. In such clinical laboratories it can be observed, on the one hand, that some of the objectives of the strategic plan (*i.e.* “extension of existing technologies and procedures for biomedical diagnosis”) may be included in a single perspective (*i.e.* internal process), but is carried out in all Departments. On the other hand, other objectives of the strategic plan (*i.e.* “reorganization of a department”) may be directed to a single department, but initiatives to achieve the objective may be included in all perspectives.

### 2. Design of the strategic map of the perspective’s objectives.

The map is developed analysing the cause-effect relationships of the different objectives among them. It represents, in graphic form, how the objectives are linked within their perspective, and also their relationships between the four perspectives, constituting what is called the alignment of the objectives. This alignment helps the coherence between them to be understood and shows how the achievement of some objectives leads to the achievement of others in the form of a cascade. This strategic map begins with the human factor, in terms of learning objectives, and then finally develops into the customer objectives.

### 3. Definition of indicators and goals of the perspective’s objectives.

In order to monitor the perspective objectives, relevant indicators and goals have to be designed which will allow the results obtained to be displayed in a quantitative manner. In this regard, it should be noted that the results of some indicators may assess more than one objective, whether or not it belongs to the same perspective. Moreover, in defining the indicators, the organizational characteristics of the clinical laboratory (if it has autonomy of management or a system of promotion of professionals, among others) and its type (if it is a reference clinical laboratory or not) have to be taken into account. For better management indicators should show, in a clear and understandable way, to the whole organization the contribution of the initiatives defined each year in the achievement of the strategic plan.

The results of the indicators, defined in the customer perspective, have to measure the intangible aspects of the organization as those that demonstrate the capacity of its professionals to advise any client or user when they performs a consultation. As well as, they must show the ability of the clinical laboratory’s Managing Board to respond to complaints and incidents that may arise from laboratory performance. In the case of a reference clinical laboratory, indicators should communicate the ability to make the laboratory into a leader based on the excellence of its activity.

One important aspect to consider is the fact that the results of the objective’s indicators of the BSC’s financial perspective must be able to demonstrate, to the entire organization, the leadership capacity of the clinical laboratory’s Managing Board to achieve budget compliance. Moreover, the ability to generate sufficient self-financing will enable them, on the one hand, to invest in those technological resources necessary for a better service to citizens and, on the other hand, to increase the training of all its professionals.

On the basis of the results obtained, indicators from the internal process perspective have to demonstrate the improvement of diagnostic and analytical process efficiency. Results of these indicators can also demonstrate the greater efficacy of the analytical process that must be reflected in a better compliance with the turnaround time and in obtaining higher quality results. In a reference clinical laboratory it should also show its capacity to innovate laboratory medicine.

The results of the learning perspective’s indicators, in conjunction with those from the perspective of internal processes, must allow the Managing Board to know at all times the dedication it has, both to overcome the resistance to change, and to increase the professional competence of personnel in responding to new orientations that the clinical laboratory will incorporate.

Once the BSC is designed, it is important to inform the laboratory professionals of the objectives defined. Moreover, as the first results of the indicators are obtained, they must be shown to these professionals, with values that clearly demonstrate both their degree of participation and the achievements derived from their activity. This will synergistically orient their capacity and efforts with those of the Managing Board in order to obtain the achievements of the clinical laboratory.

## Application of the BSC to assess the four year strategic plan in a reference clinical laboratory

The organizational structure of the clinical laboratory located in Catalonia (Spain) includes the Departments of the different specialties of the laboratory (Pathology, Clinical Biochemistry, Haematology, Immunology and Microbiology). In addition to these Departments, there are two Operational Areas where automated activity is performed, both in equipment for analysis based on molecular absorption spectrophotometry and in immunological techniques (Core Laboratory), as well as in equipment for carrying out studies based on molecular biology (Core Molecular Biology). It also has six transversal support units: client management, quality, economic-administrative management area, teaching, research and information systems coordination. The activity performed in the clinical laboratory each year, more than 7.000,000 determinations or diagnostic studies from a catalogue of more than 2800 tests, is carried out by 430 professionals. It is also a reference center for almost 300 laboratories in Spain which send their specialised tests to perform. Moreover, in this clinical laboratory different population programs are carried out, such as newborn screening in the detection of congenital conditions, prenatal screening tests, histocompatibility studies for solid organ and stem cell transplantation (*e.g.* HLA typing and viral serologies for all organ transplants) and program for early detection of colorectal cancer in Catalonia.

It has to be stated that the elaboration of a BSC was facilitated because laboratories have implemented quality management systems (both ISO 9001 and ISO 15189), which allow the Managing Board of the clinical laboratory to acquire greater knowledge of the organization and its context. Moreover, the Managing Board uses the annual review report of the quality management system to know about the improvement actions that are needed to be implemented, as well as the annual initiatives that are required (*18*).

The perspective objectives established for this clinical laboratory, whose vision is to lead laboratory medicine generating and integrating new knowledge and technological changes and be recognized as a clinical laboratory of excellence, are shown in Table 1. In these theoretical objectives, the annual initiatives defined to achieve the objectives of the four-year strategic plan are classified. The strategic plan of the laboratory was elaborated taking into account the guidelines of the Managing Board of the Hospital. Moreover, there is an alignment between both strategic plans whose priority areas are: patients (the reason of being of the Hospital); professionals (their engine) and resources (which make continuity and innovation possible in care).

**Table 1:** Perspective objectives

**Perspective**	**Objectives**
Customers	1. To increase customer satisfaction
	2. To improve the image and prestige of the laboratory
	3. To improve patient safety
	4. To meet the health needs and expectations of the population
Financial	1. Compliance with the strategic budget
	2. Generation of sufficient self-financing to meet the objectives of the laboratory mission
Internal Process	1. To innovate laboratory medicine
	2. To improve the diagnostic and process efficiency
	3. To improve the quality and efficacy of the process and product
Learning	1. To motivate the personnel
	2. To increase the training of strategic personnel
	3. To increase staff competence
	4. To increase internal communication

**Figure 1:**
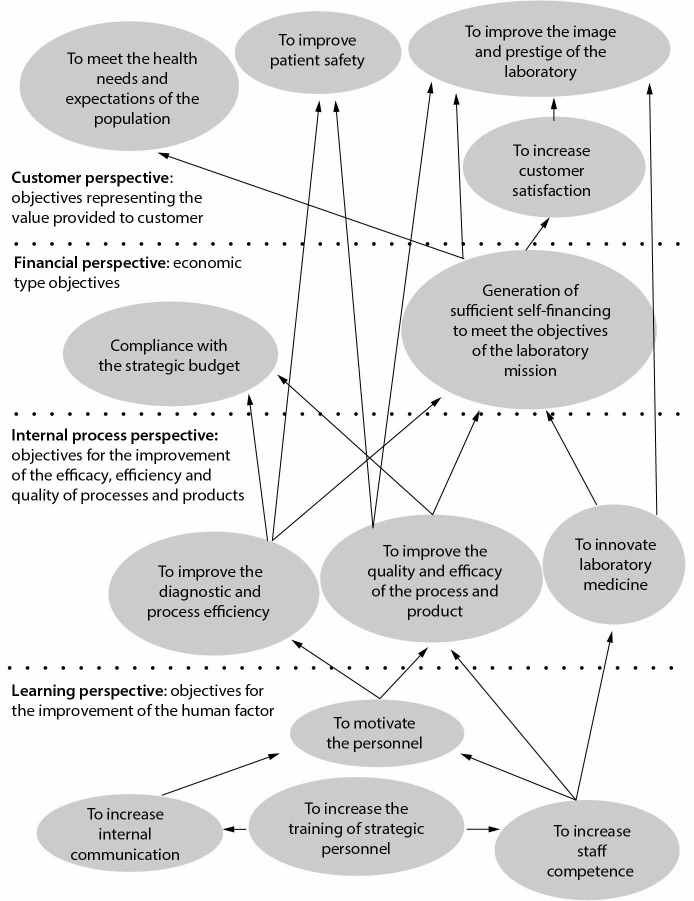
Strategic map of perspective objectives

In Figure 1 the relationship between the objectives of the same perspective and the relationships of the objectives between the four perspectives are shown.

The indicators and goals of the objectives of each perspective are shown in Table 2.

**Table 2:** Indicators and goals of the customer perspective objectives

**Objectives**	**Indicators**	**Goals**
**Customer perspective**		
1. To increase customer satisfaction	Degree of satisfaction internal and external customers (assessment of treatment, reliability of results and overall evaluation)	NSI > 75
	Number of complaints received from users	≤ 12 / year
2. To improve the image and prestige of the laboratory	Degree of external client satisfaction for the advice received in case of consultation	NSI > 75
	Number of publications in scientific journals with impact factor	≥ previous year
3. To improve patient safety	Number of incidents reported by Hospital staff due to laboratory performance	≤ previous year
	Percentage of turnaround time compliance	> 95%
4. To meet the health needs and expectations of the population	Percentage of population programs that are carried out in the laboratory	≥ previous year
	Percentage of tests included in catalogue addressed to the oncological patient in relation to all the incorporated tests	≥ 10%
**Financial perspective**		
1. Compliance with the strategic budget	Percentage of deviation of the budget in consumables material, personnel and total budget	0 - 1%
2. Generation of sufficient self-financing to meet the objectives of the laboratory mission	Annual variation in the number of patients and requests received in the laboratory	0 - 1%
	Annual variation in the billing to external clients	0 - 1%
	Customer loyalty: the percentage variation of billing to clients who requested analysis to the laboratory in the last two years	10%
	Annual variation in the number of clients: percentage of new clients and percentage of lost clients	0 - 5%
**Internal process perspective**		
1. To innovate laboratory medicine	Percentage of tests incorporated in the Catalog in response to new areas of knowledge in relation to all incorporated tests	> 10%
2. To improve the diagnostic and process efficiency	Percentage of tests incorporated to increase the diagnostic efficiency thanks to technological development of the total test incorporated	> 10%
3. To improve the quality and efficacy of the process and product	Percentage of determinations with defaults that do not meet the turnaround time every month of the year	< 3%
	Percentage of results of external quality assurance programs to be reviewed according to the criteria of the organizer	< 3.5%
	Percentage of annual initiatives achieved	> 80%
**Learning perspective**		
1. To motivate the personnel	Degree of professional satisfaction (the assessment of actions dedicated to improve their quality of life and human and professional promotion)	NSI > 75
	Percentage of personnel who have improved their professional category	> 10% triannual
	Percentage of absenteeism	< 2%
2. To increase the training of strategic personnel	Number of training activities aimed at professionals who carry out strategic activities	> 1 / year
3. To increase staff competence	Percentage of specific training activities to increase professional competence	> 10%
	Percentage of technicians in a laboratory who can develop the same activity with respect to the total number of technicians	> 20%
4. To increase internal communication	Number of actions carried out to increase the internal communication	> 3 / year
NSI - normalized satisfaction index. An NSI between 75 and 85 reflects a good valuation and more than 85 an excellent valuation.		

Furthermore, that indicators have to be defined taking into account the objectives and goals of the Government of the country. In this way, since 2005 in Catalonia, patient safety is one of the main priorities of the Ministry of Health of the Government’s objectives. So, in the proposed BSC those aspects in which the performance of the clinical laboratory can give rise to incidents in other points of the Hospital, or those caused by the delay in the delivery of a result or a diagnostic study, are especially assessed.

As it can be seen in Table 2, some of the goal’s values are expressed as NSI (Normalized Satisfaction Index) which is obtained from a calculation developed by International Business Machines Corporation (IBM) that is based on the penalty of the lowest scores.

NSI = [(Ax0) + (Bx25) + (Cx50) + (Dx75) + (Ex100)] / N

Where: A = number of responses with a satisfaction score of 1 (very bad), B = number of responses with a satisfaction score of 2 (bad), C = number of responses with a satisfaction score of 3 (regular), D = number of responses with a satisfaction score of 4 (good), E = number of responses with a satisfaction score of 5 (very good) and N = A + B + C + D + E.

The organizational structure of this reference clinical laboratory, that it is based on management autonomy, allows the Managing Board to promote, on the one hand, efficiency in the management of human and technological resources and, on the other, the dedication of resources to special tests, which are in development or that are not requested frequently. That is why the objectives of the strategic plan and its annual initiatives are aimed at implementing the most cutting-edge services using the latest technology. Sometimes this includes the use of the latest large equipment which allows the automated generation of a large number of results, or the use of innovative technologies not generally implemented in the network of healthcare clinical laboratories. It is important to note that the results of the indicators of the financial perspective allow the efficiency of the clinical laboratory in the budget management approved by the Hospital Managing Board to be demonstrated, both in the costs of consumables and equipment, as well as personnel. Likewise, the greater efficacy of all internal processes, especially the strategic planning of the organization and personnel management processes, will be shown in the achievement of a larger number of the initiatives defined annually.

Furthermore, in this reference clinical laboratory, it is important to consider the results of the learning perspective indicator, “Percentage of professionals who have improved their professional category”, because they will reflect the effort made by all the laboratory’s personnel to maintain and improve their competence and their participatory and collaborative spirit.

## Concluding remarks

A comprehensive BSC that includes theoretical objectives for each perspective, as well as indicators and the goals to be achieved, can be a valid, manageable and simple tool in the clinical laboratory to monitor and quantitatively measure the degree of achievement of the objectives of a strategic plan in those organizations that, due to their complexity, have numerous objectives and each year define a large number of very different initiatives.

This model of BSC has the particularity of having theoretical objectives of perspectives rather than the original strategic objectives. It is adaptable to any organizational model and to the resources available. Yet, like all BSCs, its use allows us to demonstrate the laboratory’s strategy in carrying out its mission.

The BSC is a useful tool to demonstrate to the hospital Managing Board the effectiveness of the clinical laboratory’s managing board and professionals both in achieving the initiatives defined annually, as well as in the development of the strategic plan. Moreover, it also shows how it can fulfil its capacity to generate self-financing that allows it to carry out initiatives of greater risk oriented to customers. The annual results of the indicators allow us to identify in which perspectives the efforts have to be prioritized.
